# The G-quadruplex fluorescent probe 3,6-bis(1-methyl-2-vinyl-pyridinium) carbazole diiodide as a biosensor for human cancers

**DOI:** 10.1038/s41598-018-34378-8

**Published:** 2018-10-31

**Authors:** Ting-Yuan Tseng, Wei-Wen Chen, I-Te Chu, Chiung-Lin Wang, Cheng-Chung Chang, Mei-Chun Lin, Pei-Jen Lou, Ta-Chau Chang

**Affiliations:** 1 Institute of Atomic and Molecular Sciences, Academia Sinica, Taipei, 10617 Taiwan; 2 Institute of Biomedical Engineering, National Chung-Hsing University, Taichung, Taiwan; 3 Department of Otolarynglogy, National Taiwan University Hospital, Hsin-Chu Branch, Taiwan; 4 Department of Otolarynglogy, National Taiwan University Hospital and National Taiwan University College of Medicine, Taipei, Taiwan

## Abstract

Using time-gated fluorescence lifetime imaging microscopy, significantly more signals from 3,6-bis(1-methyl-2-vinyl-pyridinium) carbazole diiodide (*o*-BMVC) foci, characterized by the longer fluorescent decay time of *o*-BMVC, were detected in six types of cancer cells than in three types of normal cells. Accumulating evidence suggested that the *o*-BMVC foci are mainly the G-quadruplex foci. The large contrast in the number of *o*-BMVC foci can be considered as a common signature to distinguish cancer cells from normal cells. Further study of tissue biopsy showed that the *o*-BMVC test provides a high accuracy for clinical detection of head and neck cancers.

## Introduction

Cancer remains as one of the leading causes of death in many countries. Since early diagnosis can improve the survival of cancer patient, the early detection of cancer has been an unmet and urgent medical need. Thus, finding a common target would be a great advantage to prevention, diagnosis, prognosis, and treatment in personalized medicine. Considering the great diversity in the root cause of different cancers, it is very challenging to comprehend the use of a common signature for all cancer types.

G-quadruplex (G4) structures formed by the stacking of G-quartets with Hoogsteen hydrogen bonding of four guanines have gained much attention as a possible target for cancer research^1,2^. The importance of G4 formation has been associated with genome instability, genetic diseases, and cancer progression^3–6^. Recently, a high-throughput sequencing-based method has identified 716,310 potential G4s in the human genome^7^. In addition, high density of G4s was found in the promoters, 5′UTR of transcribed genes, and splicing sites, and particularly in cancer-related genes^5,6^. We have previously used G4 fluorescent probe to demonstrate the existence of G4 structures in the metaphase chromosome^8^. Biffi *et al*.^9^ have used a fluorescently labeled G4-specific antibody (BG4) to visualize the G4 foci in both cancer and normal cells. They found ~30% more in the number of G4 foci in HeLa cancer cells than in MRC-5 normal cells. Hansel-Hertsch *et al*.^6^ found that immortalized keratinocytes (HaCaT) showed ~4-fold more G4 foci than do normal human epidermal keratinocytes (NHEK) using BG4 immunofluorescence microscopy. It appears that the difference in G4 foci between cancer cells and normal cells warrants further study.

**Figure 1:**
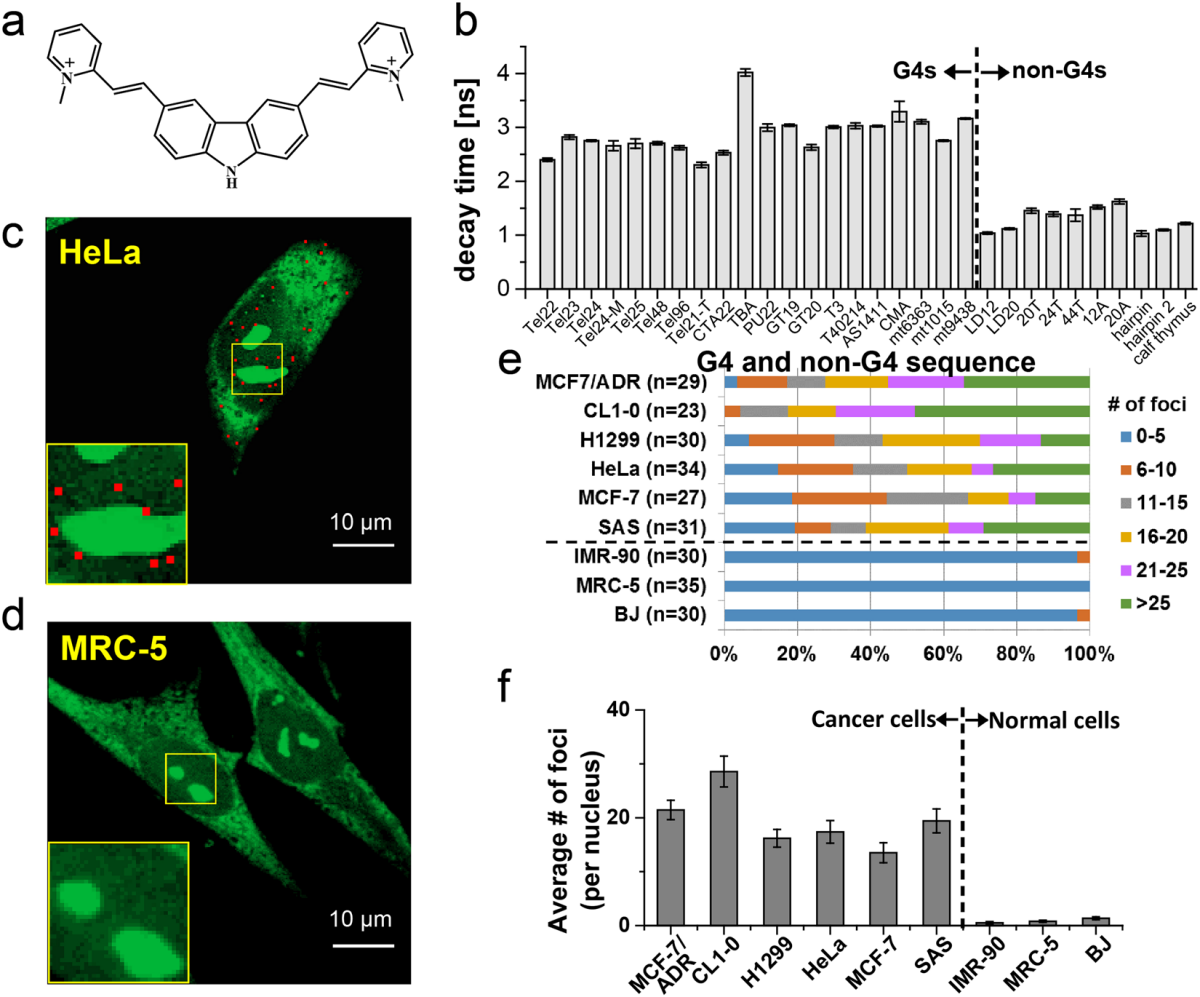
The large contrast of *o*-BMVC foci between cancer and normal cell lines. Chemical structure of *o*-BMVC (**a**). The fluorescence decay times of *o*-BMVC upon interaction with various DNA sequences in 150 mM K^+^ solution measured on a coverslip (**b**). The analyzed binary images of *o*-BMVC foci in fixed HeLa cancer cells (**c**) and in fixed MRC-5 normal cells (**d**). The analyzed binary images were separated into two colors: red (decay time ≥2.4 ns) and green (decay time <2.4 ns). Quantitative analysis of the number of *o*-BMVC foci in the nucleus of various cell lines, where n is the number of cells (**e**). The average number of G4 foci per fixed cell from various cell lines (**f**).

Bioimaging is a powerful tool in visualizing cellular responses and monitoring target/probe interaction in cells^10,11^. Using imaging, fluorescent G4 ligands could provide a relatively simple, low-cost, and rapid approach to visualize G4s in cells. Among the limited available G4 fluorescent probes^12,13^, a 3,6-bis(1-methyl-2-vinylpyridinium) carbazole diiodide (*o*-BMVC) molecule has shown a higher binding affinity to G4 DNA than to duplex DNA by nearly two orders of magnitude^14^. The chemical structure of *o*-BMVC was shown in Fig. 1a. Of importance is that fluorescence lifetime imaging microscopy (FLIM) showed longer fluorescent decay times of *o*-BMVC upon interaction with most G4s formed by G-rich sequences in telomeres and some promoter oncogenes (≥2.4 ns) than with other structures such as linear duplexes and hairpin structures (~1.2 ns)^14^. Using time-gated FLIM, an image of stained cells can be derived from two components (longer and shorter decay times) separated by a threshold decay time. Here, we use time-gated FLIM of *o*-BMVC to study the difference in G4s in fixed cells from various cell lines, particularly for the comparison of cancer and normal cells. The finding of many more numbers of *o*-BMVC foci in the nuclei of six cancer cell lines than three normal cell lines prompted us to validate these findings in clinical samples.

## Results

### *o*-BMVC foci can act as a common biosensor of cancer cells

We have used FLIM to measure the fluorescence decay time of *o*-BMVC upon interaction with 32 DNA sequences. All sequences were listed in Supplementary Table S1. The results showed that the decay times of *o*-BMVC are longer upon interaction with 20 G4s than with 10 sequences of duplex and single stranded DNA (Fig. 1b). Of interest was that one of the G-rich sequences, TBA-3G, could form a G-triplex with two G:G:G triad planes^15,16^, while one of the non-G-rich sequences, HD28, could form a triplex containing both T:A-T and C^+^:G-C triplets^17^. The FLIM results showed that the fluorescence decay time of *o*-BMVC upon interaction with TBA-3G was 3.1 ns, which was longer than ~1.5 ns upon interaction with HD28 but was similar to the decay time upon binding to G4s. Likewise to the G4 formation, a G-triplex is also a noncanonical DNA structure formed by the stacking of G-triplets. The G-triplet was made by Hoogsteen hydrogen bonds between guanine residues to stabilize G:G:G triad plane. Given that external stacking to the G-quartets is the major binding of *o*-BMVC to G4^14^, the 3.1 ns decay time measured from *o*-BMVC upon interaction with TBA-3G is also likely due to external stacking to the G-triplets.

Given that TBA-3G was originally proposed as G4 folding intermediates of the TBA^18^, TBA-3G truncated from TBA was used to verify the presence of G-triplex^15,16^. Such G-triplex was also studied in human telomere^19,20^. Although limited G-triplexes have been documented, it is possible to have abundant G-rich sequences with 3 G-tracts to form G-triplex structures in human genome. Thus, more studies are necessary to establish the importance of G-triplex formation, such as the possible contribution from G-triplexes to the *o*-BMVC foci in distinguishing cancer cells from normal cells. Considering that the G4 structures are more stable than the G-triplex structures (Supplementary Fig. S1) and the G4s are potential targets for cancer research, we therefore put our attention on the contribution from G4s to the *o*-BMVC foci in this work.

Confocal microscopy showed that *o*-BMVC is rapidly taken up and is mainly found in the cytoplasm of live cells^10^, while it can enter the nucleus of fixed cells. Typical FLIM images of fixed HeLa cancer cells and fixed MRC-5 normal cells incubated with *o*-BMVC were shown in Supplementary Fig. S2. For simplicity, we divided the image into two temporal regions by a decay time at 2.4 ns as a threshold. Using the Otsu threshold method^21^, the FLIM images were analyzed and separated into two channels: red (decay time ≥2.4 ns) and green (decay time <2.4 ns) (Supplementary Fig. S3). Time-gated FLIM allowed us to visualize the difference in the number of *o*-BMVC foci between fixed cancer cells and fixed normal cells. The analyzed binary images showed the detection of tens of *o*-BMVC foci in HeLa cancer cells (Fig. 1c). By contrast, few *o*-BMVC foci were detected in MRC-5 normal cells (Fig. 1d). The red spots resulting from *o*-BMVC staining are named *o*-BMVC foci, which are most likely due to interaction with a cluster of G4s within a single optically resolvable area. The same approach was also applied to other cell lines, including BJ and IMR-90 normal cell lines together with MCF-7, H1299, CL1-0, SAS, and MCF-7/ADR cancer cell lines (Supplementary Fig. S4). Significantly more numbers of *o*-BMVC foci detected in cancer cells than in normal cells suggested that the number of *o*-BMVC foci (≥8) could be an eligible signature for cancer cells.

The detection of *o*-BMVC foci in the cytoplasm may be due to *o*-BMVC interaction with RNA G4s^22,23^, mitochondrial DNA G4s^24^, or other unknown substrates in cancer cells. Given that the majority of DNA are in the nucleus, here quantitative analysis of *o*-BMVC foci in the nuclei also showed significantly more numbers of *o*-BMVC foci in cancer cells than in normal cells (Fig. 1e). In addition, the average numbers of *o*-BMVC foci detected in the nuclei of these cell lines were summarized in Fig. 1f. We then used DNase and RNase to examine whether the major target of *o*-BMVC is oligonucleotides. The fluorescence of *o*-BMVC increases >80-fold upon interaction with G4s and *ca*. 20-fold upon interaction with calf thymus DNA^14^. Confocal microscopy showed very weak *o*-BMVC fluorescence in the HeLa cancer cells pre-treated with both DNase and RNase (Supplementary Fig. S5a), indicating that *o*-BMVC foci are mainly due to binding of *o*-BMVC to oligonucleotides. In addition, the use of DNase pretreatment indeed markedly reduced the number of *o*-BMVC foci in HeLa cancer cells (Supplementary Fig. S5b). Considering that *o*-BMVC foci are characterized by the long fluorescence decay time, we assumed that the *o*-BMVC foci are mainly the G4 foci.

### *o*-BMVC foci are likely G4 foci

**Figure 2:**
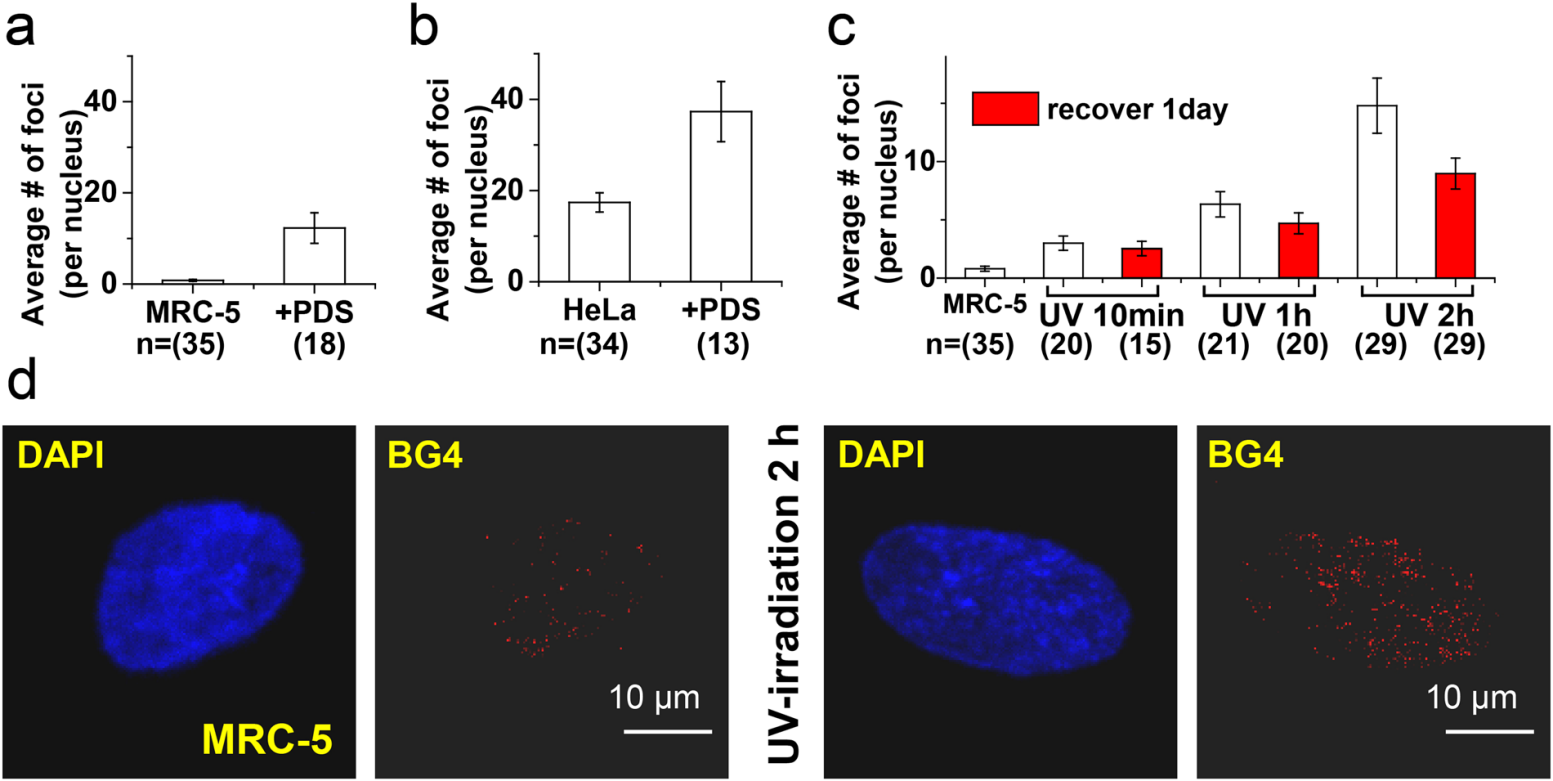
*o*-BMVC foci induced by PDS or UV- irradiation. Quantitative analyses of the number of *o*-BMVC foci in the pre-treated MRC-5 normal cells (**a**) and in the pre-treated HeLa cancer cells (**b**) without and with PDS. Quantitative analyses of the number of *o*-BMVC foci without UV irradiation and time-dependent UV irradiation together with the UV treated cells in the dark overnight (Red bars) (**c**). Confocal images of MRC-5 without (left) and with (right) exposure to UV-irradiation and stained by DAPI and BG4 antibody (**d**).

Rodriguez *et al*.^25^ found that pyridostatin (PDS), a G4 ligand, not only induced DNA damage at sites enriched in G4 motifs but also affected the expression of genes that have G4-forming sequences within their promoters. They further visualized G4 foci in simian virus (SV40)-transformed MRC-5 human fibroblasts by optical imaging with Alexa Fluor 594 labeled PDS. Consistent with their finding, our quantitative analyses showed that marked increase in the number of *o*-BMVC foci was detected in PDS pretreated MRC-5 normal cells (Fig. 2a). Here the use of PDS without SV40 transformation could increase the number of G4 foci in MRC-5 normal cells. In addition, Biffi *et al*.^9^ detected 2.9-fold more G4 foci in the PDS pretreated human U2OS cancer cells using the BG4 antibody. Consistent with their finding, we found that PDS increased the number of *o*-BMVC foci by ~2-fold in the nuclei of HeLa cancer cells (Fig. 2b). The agreement of these two approaches with the use of BG4 antibody and *o*-BMVC molecule as G4 sensors independently supported that the *o*-BMVC foci are mainly the G4 foci.

DNA damage can lead to genomic instability, opening the chromatin, increasing the risk of exposing unprotected G4 site. The finding of large numbers of *o*-BMVC foci induced by PDS in both cancer and normal cells is important to illustrate that more opening of the total chromatin resulting from DNA damage could facilitate G4 formation from unprotected G-rich sequences. Thus, we hypothesized the more open the chromatin, the more detection the number of *o*-BMVC foci in cells. Given that UV-irradiation can damage DNA by triggering loss of chromatin integrity, pretreatment with DNA damage by exposing MRC-5 normal cells to UV light showed marked increases in the number of *o*-BMVC foci as a function of the UV-irradiation time (Fig. 2c), which supported our hypothesis. Moreover, it is important to find that the number of *o*-BMVC foci induced by UV light showed appreciable decrease after the UV light-treated cells kept in the dark overnight (Fig. 2c). Further study by using BG4 antibody confirmed that DNA damage induced by UV-irradiation could generate more numbers of G4 foci in MRC-5 cells (Fig. 2d). This finding also supports that the *o*-BMVC foci are mainly the G4 foci.

### Validation of *o*-BMVC foci for clinical screening of human cancers

**Figure 3:**
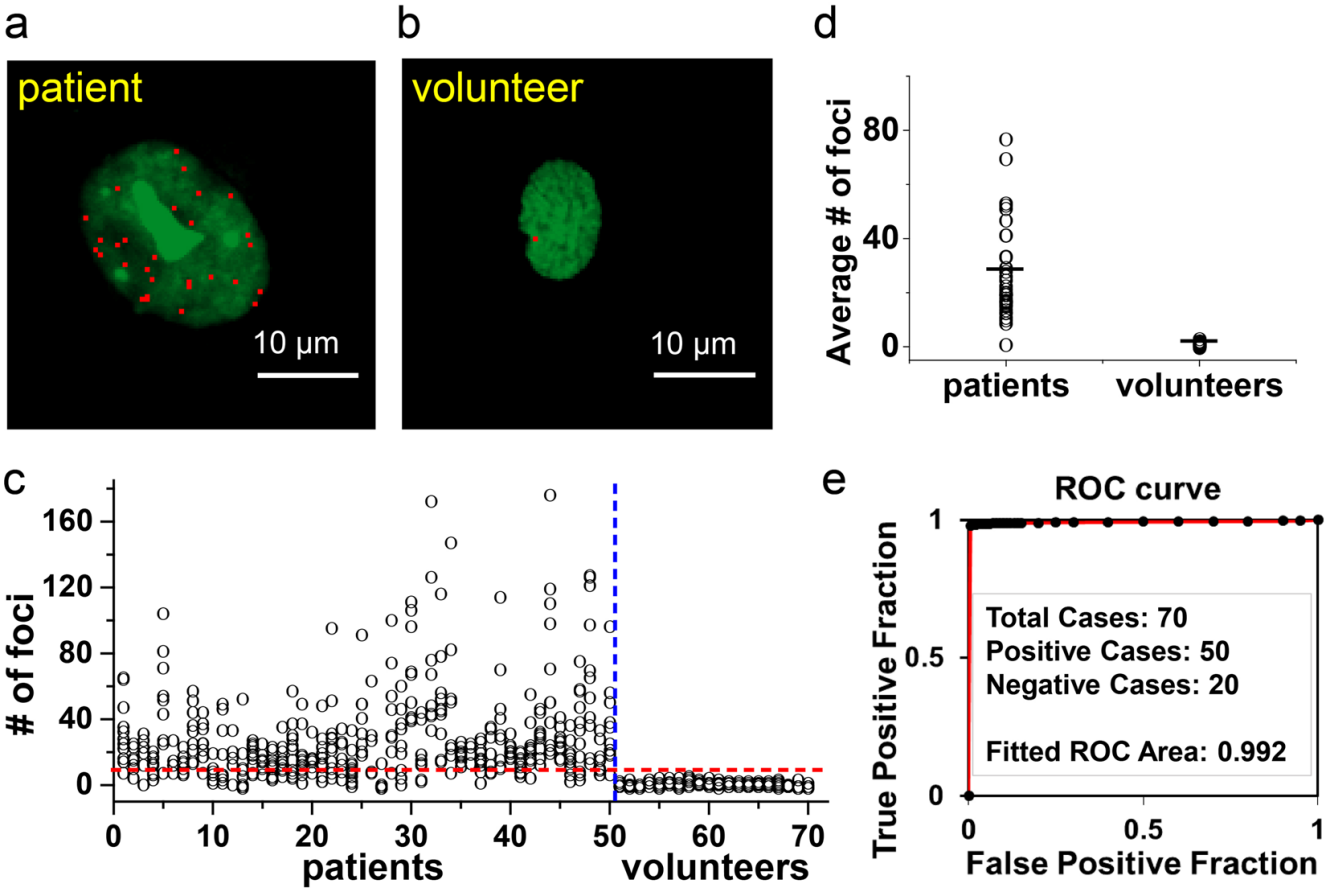
*o*-BMVC foci in tissue biopsy. Representative analyzed binary images of *o*-BMVC stain of a tongue squamous cell carcinoma cell (**a**) and a buccal epithelial cell (**b**). Quantitative measurements of the number of *o*-BMVC foci in ~10 nuclei for each person with 50 patients and 20 volunteers were plotted (**c**). The average number of *o*-BMVC foci of each person (**d**). The receiver operating characteristic (ROC) curve (**e**).

To test the clinical validation of *o*-BMVC foci in tissue biopsy, a total of 50 head and neck cancer (HNC) samples obtained during surgery and 20 normal oral samples collected from healthy volunteers were examined. Figure 3a showed an analyzed binary image of *o*-BMVC staining nucleus of a tongue squamous cell carcinoma sample. Figure 3b showed an analyzed binary image of *o*-BMVC stain of a sample collected by a Q-tip to brush the buccal mucosa of a healthy volunteer. Quantitative measurements of the number of *o*-BMVC foci in ~10 nuclei for each person were conducted and the results of 50 patients and 20 volunteers were plotted in Fig. 3c. All the results from *o*-BMVC test together with those from cytologic examination were listed in Supplementary Table S2. Consistent with the finding in the study of cell lines, *o*-BMVC foci are hardly detectable in the normal oral epithelial cells. The average numbers of *o*-BMVC foci in tumors and in normal oral epithelial cells are 28.3 and 2.2, respectively (Fig. 3d). According to the number of *o*-BMVC foci results and the receiver operating characteristic (ROC) curve analysis, a threshold value of 7.5 was applied to differentiate malignant and benign specimens. Astonishingly, the ROC curve showed the area under curve (AUC) was 0.992 (Fig. 3e), indicating that this method provides a very high accuracy for detection of HNC cells from patients.

In addition, we found that 12 of the 16 cases with high average number of *o*-BMVC foci had stage 4 disease, 1 had stage 3, 1 had stage 2, and the remaining 2 had stage 1 disease. The 2 patients that had the highest average number of *o*-BMVC foci were one with stage 4 nasopharyngeal cancer (double cancer, the sample was from the tongue) and one with stage 1 tongue cancer but recurred with stage 4 disease. It is probably that the average number of *o*-BMVC foci correlates with the tumor burden (disease stage), which deserves further studies.

HNC is the sixth most common cancer in the world^26^. Most HNC patients are found to have a locally advanced or metastatic tumor at the time of diagnosis. Despite the improvement of multidisciplinary treatment during the past decades, the survival of locally advanced HNC patients has not been improved^27^. On the other hand, for early stage HNC patients, the treatment outcomes are generally good^28^. It is thus important to develop better screening programs and novel diagnostic measures for early diagnosis of HNC to improve the survival of patients. In this study, we used HNC tumor tissues for validation of *in vitro* cell line findings is due to both tumor and control samples are easily accessible. Furthermore, this pilot study provides the possibility that *o*-BMVC test may be used for oral swabs in the minimally invasive diagnosis of oral cancers. Further large scale clinical trials are needed to verify this point.

## Discussion

In this work, we used a G4 fluorescent probe of *o*-BMVC to stain fixed cells and found a large contrast in the number of *o*-BMVC foci between cancer cells and normal cells in time-gated FLIM images for the discrimination of human cancers. Our results are consistent with the previous study by using BG4 immunofluorescence microscopy. Biffi *et al*.^29^ showed that the use of BG4 antibody could stain G4 DNA in patient-derived tissues using immunohistochemistry. They observed that there are a greater number of G4 foci in human cancers of the liver and stomach as compared to background non-neoplastic tissue. Accordingly, it is likely that the large contrast in the number of G4 foci between cancer cells and normal cells may act as a common biomarker for human cancers.

At present, we do not know exactly why there are more numbers of G4 foci in cancer cells than in normal cells. Given that loss of genomic integrity is a common hallmark of cancer^30,31^, we anticipated that the loss of chromatin integrity also plays an active role in the predominance of G4 foci in cancer cells because of either more G4 formation or easier for *o*-BMVC binding. Of importance is the gradual decrease of the induced *o*-BMVC foci after terminating UV-irradiation, suggesting that the G4 foci induced by DNA damage are different from the G4 foci detected in cancer cells. It is likely that the decrease of *o*-BMVC foci is due to the function of DNA repair. Considering impaired DNA repair is commonly occurred in many cancers^32^, this mechanism may play a major role in enriching the G4 formation in cancer cells.

Given that the etiology and clinical manifestation of different cancers are widely varied, direct evidence to confirm that the *o*-BMVC foci are the G4 foci is critical to validate the use of G4 foci as a common biomarker of cancer cells. To explore this possibility, one can determine the G4 sequences by gene sequencing and NMR and identify the surrounding proteins by mass analysis from isolated *o*-BMVC foci in cancer cells. Such study may shed new insights into G4 formation, identify mechanisms of G4 function, and unravel therapeutic targets of G4 related genes. Moreover, the determination of gene sequence can verify the potential contribution to the *o*-BMVC foci from the G-triplexes formed by the G-rich sequences with three G-tracts in human genome. At present, we are limited by the techniques to micro-dissect and to collect sufficient isolated *o*-BMVC foci. Nevertheless, the *o*-BMVC foci provide an opportunity for direct approach in verifying the use of G4 foci as a common cancer biomarker.

In summary, the significance of this study was to unequivocally demonstrate that many more *o*-BMVC foci are present in cancer cells than in normal cells. Although our tested cells are only small amounts of the vast cell lines, this finding lays the foundation for the development of *o*-BMVC foci as a common indicator of cancer cells. Consistent with the finding in the study of cell lines, the average numbers of *o*-BMVC foci are 28.3 from 50 head and neck patients and 2.2 from 20 healthy volunteers. Our findings suggest that the *o*-BMVC test is a novel assay for clinical screening of human cancers.

Quantitative measurement of *o*-BMVC foci is useful to establish a platform in monitoring cellular response under different conditions. For instance, DNA damage can induce the *o*-BMVC foci, while DNA repair may reduce the *o*-BMVC foci, implying that DNA repair plays a role in the large contrast of *o*-BMVC foci between cancer and normal cells. In addition, *o*-BMVC foci may serve as a convenient indicator to monitor carcinogenic transformation, which is essential to the development of early screening of cancer. Visualizing the change of *o*-BMVC foci may also lead to yield new insights into the underlying mechanisms of carcinogenesis and provide a new direction for early diagnosis and drug development in personalized medicine and biomedical research.

## Methods

### Chemical and sample preparation

The synthesis of *o*-BMVC can be found elsewhere^14^. All oligonucleotides purified by HPLC were purchased from Biobasic Inc. (Canada). Solutions of 10 mM Tris-HCl (pH 7.5) and 100 mM KCl mixed with each oligonucleotide were heated to 95 °C for 5 min, cooled slowly at 1 °C/min to room temperature and then were stored overnight at 4 °C before use. The concentration of each oligonucleotide was determined by UV absorption nanophotometry (Implen, Germany).

### Cell cultures

CL1-0, a human lung carcinoma cancer cell line, was kindly provided by Prof. P.-C. Yang (National Taiwan University). MCF-7/ADR, a multidrug-resistant human breast cancer cell line, was kindly provided by Prof. Y.-H. Chen (National Taiwan University). SAS, a human tongue carcinoma cancer cell line, was kindly provided by Prof. J.-S. Chia (National Taiwan University College of Medicine). The human normal lung fibroblast cell line MRC-5, human normal foreskin fibroblast cell line BJ, human normal lung fibroblast cell line IMR-90, human cervical adenocarcinoma cell line HeLa, human lung carcinoma cell line H1299 and human breast cancer cell line MCF-7 were obtained from the American Type Culture Collection (ATCC). CL1-0 and H1299 cells were cultured in RPMI1640 medium supplemented with 10% fetal bovine serum (FBS) and 1% antibiotics. MCF-7/ADR and SAS cells were cultured in DMEM medium supplemented with 10% FBS and 1% antibiotics. HeLa, MCF-7, MRC-5, BJ and IMR-90 cells were cultured in MEM medium supplemented with 10% FBS and 1% antibiotics. All cell lines were cultured in 5% CO_2_ at 37 °C. The antibiotic concentration was 100 U/mL penicillin and streptomycin.

### Confocal microscopy

For the study of DNase and RNase pretreatment, HeLa cells were fixed with 70% ethanol for 10 min on coverslip and were treated with 20 μg/ml of DNase and RNase for 1 h at 37 °C followed by 5 µM *o*-BMVC staining for 10 min. Fluorescence excitation was carried out at 470 nm for *o*-BMVC.

### Immunofluorescence

For immunofluorescence, MRC-5 cells were grown on glass coverslips without and with exposure to UV-irradiation and then were fixed with methanol/acetic acid (3:1) for 10 min. Fixed cells were permeabilized with 0.2% Triton-X100 and then were blocked with 2% Bovine serum albumin (BSA). After blocking, cells were incubated with a commercial BG4 antibody (Ab00174-1.1; absolute antibody, UK) for 1 h and then were incubated with anti-mouse Alexa Fluor 647-conjugated (A21235, Invitrogen, USA) secondary antibodies for 1 h. Samples were co-stained with 0.1 μM DAPI for 20 min and were visualized using a confocal microscope (Leica TCS SP8). Fluorescence excitation was carried out at 647 nm for Alexa Fluor 647 and 405 nm for DAPI.

### Fluorescence Lifetime Imaging Microscopy (FLIM)

The setup of the FLIM system consisted of a picosecond diode laser (laser power, 5 mW) with an emission wavelength of 470 nm (LDH470; PicoQuant, Germany) and a ~70 ps pulse width for the excitation of *o-*BMVC under a scanning microscope (IX-71 and FV-300; Olympus, Japan). The fluorescent signal from of *o*-BMVC was collected using a 60× NA = 1.42 oil-immersion objective (PlanApoN; Olympus, Japan), passing through a 550/88 nm bandpass filter (Semrock, USA), followed by detection using a SPAD (PD-100-CTC; Micro Photon Devices, Italy). The fluorescence lifetime was recorded and analyzed using a time-correlated single-photon counting (TCSPC) module and software (PicoHarp 300 and SymPhoTime v5.3.2; PicoQuant, Germany). FLIM images were constructed from pixel-by-pixel lifetime information.

For the study of fixed cells, cells on coverslip were fixed with 70% ethanol for 10 min and then stained with 5 µM *o*-BMVC for 10 min at room temperature. For the study of PDS pretreatment, HeLa and MRC-5 cells on coverslip were treated with 1 µM PDS overnight. After washing twice, cells were fixed with 70% ethanol for 10 min and then stained with 5 µM *o*-BMVC for 10 min at room temperature. For the study of DNase treatment, HeLa cells on coverslips were fixed with 70% ethanol for 10 min and then treated with 20 µg/ml DNase for 1 h at 37 °C followed by 5 µM *o*-BMVC staining for 10 min.

### Quantitative analysis of *o*-BMVC foci

Since the fluorescent decay time is longer upon binding to G4s than other structures, the acquired FLIM results of *o*-BMVC in cells were presented in pseudocolor and separated into two channels: white (decay time ≥2.4 ns) and red (decay time <2.4 ns) to map the G4s. Here we used HeLa cancer fixed cells as an example (Fig. S3A, left). After excluding the non-signal pixels (intensity = 0), the gray-level histogram of the longer lifetime channel can be fit as the mixture of Gaussians (Fig. S3B). The optimal threshold was further determined by the Otsu algorithm to eliminate the weaker signals, which may be due to the loose binding of G4 DNA or the non-specific binding of small cell fragments, in the longer lifetime channel. The Otsu threshold method^21^ was used to find an optimal threshold (*T*_*opt*_) to separate two clusters or the mixture of Gaussians, with the following formula:

$${T}_{opt}=argmax\{\frac{P(T)[1-P(T)]{[{m}_{f}(T)-{m}_{b}(T)]}^{2}}{P(T){\sigma }_{b}^{2}(T)+[1-P(T)]{\sigma }_{f}^{2}(T)}\}$$

where *P(T)* is the cumulative probability, *m*_*b*_ *(T)* is the mean of the background, *m*_*f*_ *(T)* is the mean of the foreground, *σ*_*b*_^2^*(T)* is the variance of the background and *σ*_*f*_^2^*(T)* is the variance of the foreground. After applying the Otsu threshold method, the weak signals can be eliminated, while the stronger signals (the red spots in Fig. S3A, right) can be preserved. The same imaging process and analysis were applied to the FLIM images of fixed cells and live cells. By using the algorithm for the image analysis, we can lower the possible counting errors in human eye detection and unambiguously quantify the number of foci in different cell lines.

### *ex vivo* study of clinical cells

For the study of normal oral samples, the buccal mucosa cells collected by Q-tips from healthy volunteers were fixed with 70% ethanol on microscope slides for 10 min and then stained with 5 µM *o*-BMVC for 10 min at room temperature. For the study of head and neck cancer samples obtained during surgery, the cancer cells were fixed with 70% ethanol on microscope slides for 10 min and then stained with 5 µM *o*-BMVC for 10 min at room temperature. After data collection, the receiver operating characteristic (ROC) curve was analyzed by web-based calculator^33^.

### Ethics Statement

This study was approved by the Institutional Review Board of National Taiwan University Hospital (NTUH REC No. 201304078RIND). Informed consent was obtained from all participants and/or their legal guardians. All methods were performed in accordance with the relevant guidelines and regulations.

## Electronic supplementary material

Supplementary Information
