# Supplementary material for: The G-quadruplex fluorescent probe 3,6-bis(1-methyl-2-vinyl-pyridinium) carbazole diiodide as a biosensor for human cancers
Source: Sci Rep. 2018 Oct 31;8:16082. doi: 10.1038/s41598-018-34378-8 (PMC6208391; doi:10.1038/s41598-018-34378-8)
Supplement: Supplementary file 1 — Supplementary Information [file 41598_2018_34378_MOESM1_ESM.docx]

**The G-quadruplex fluorescent probe 3,6-bis(1-methyl-2-vinyl-pyridinium) carbazole diiodide as a biosensor for human cancers**

Ting-Yuan Tseng,^1^ Wei-Wen Chen,^1^ I-Te Chu,^1^ Chiung-Lin Wang,^1^ Cheng-Chung Chang,^2^ Mei-Chun Lin,^3^ Pei-Jen Lou,^4,*^ Ta-Chau Chang,^1,*^

^1.^ Institute of Atomic and Molecular Sciences, Academia Sinica, Taipei 10617, Taiwan.

^2.^ Institute of Biomedical Engineering, National Chung-Hsing University, Taichung, Taiwan.

^3.^ Department of Otolarynglogy, National Taiwan University Hospital, Hsin-Chu Branch, Taiwan.

^4.^ Department of Otolarynglogy, National Taiwan University Hospital and National Taiwan University College of Medicine, Taipei, Taiwan.

* corresponding author

Prof. Ta-Chau Chang email: tcchang@po.iams.sinica.edu.tw

Tel: 886-2-2366-8231 Fax: 886-2-23620200

Prof. Pei-Jen Lou email: pjlou@ntu.edu.tw

Tel: 886-2-2312-3456 ext.65224 Fax: 886-2-2341-0905

**SUPPLEMENTARY INFORMATION**


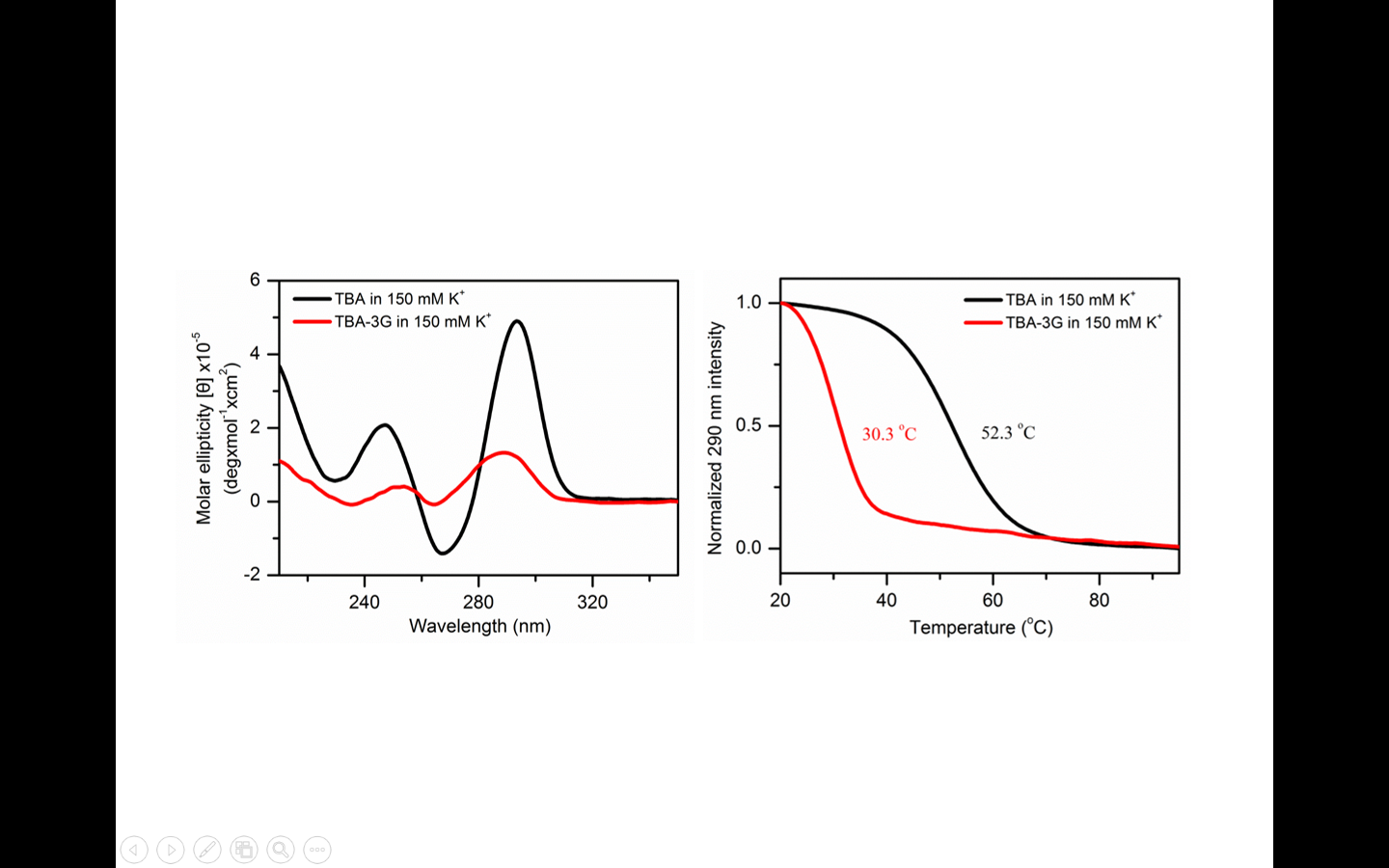


**Figure S1**. CD spectra of TBA and TBA-3G in 150 mM K^+^ solution (left) and their corresponding CD melting curves monitored at 290 nm (right).


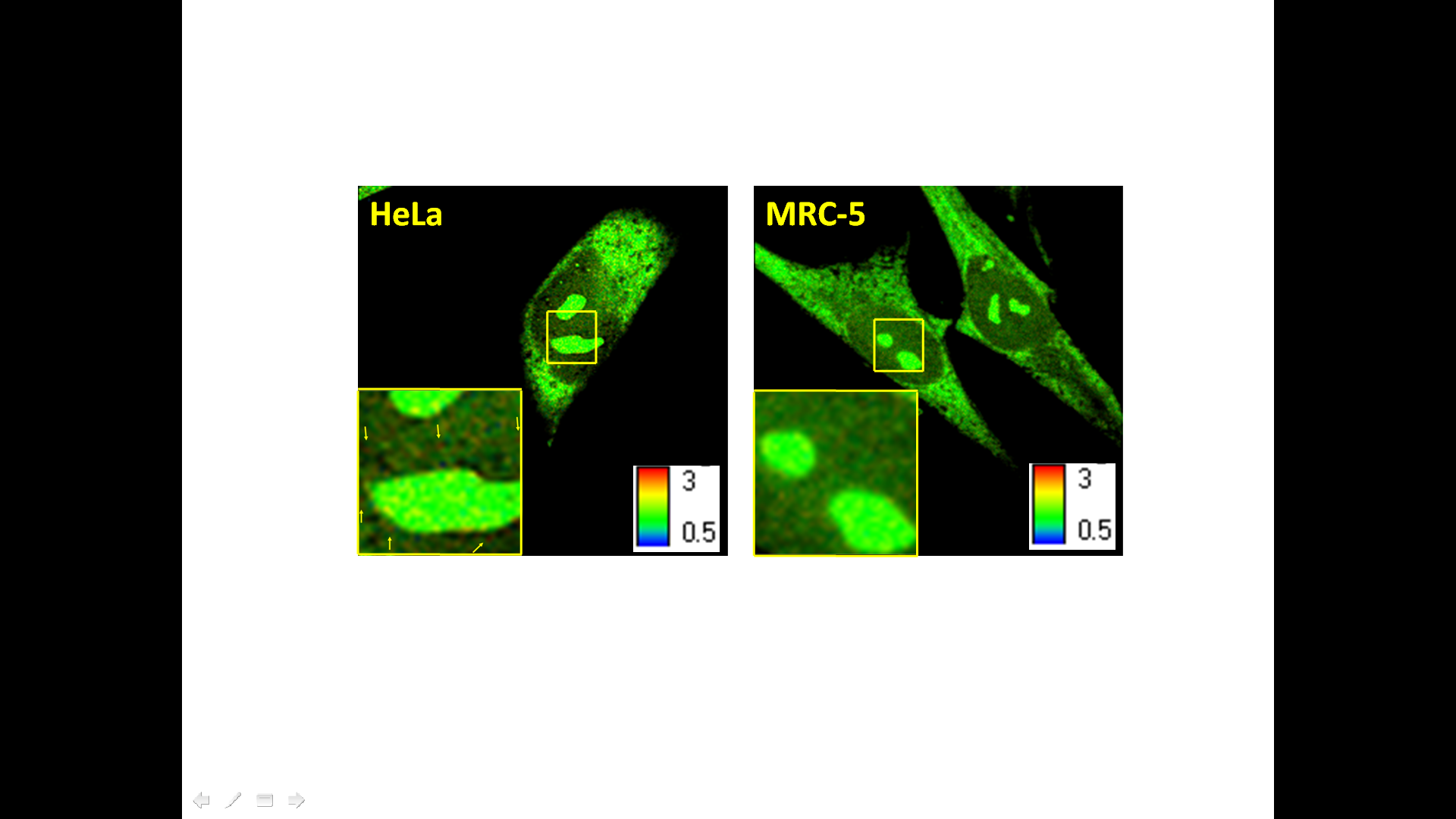


**Figure S2.** FLIM images of fixed HeLa cancer cells (left) and fixed MRC-5 normal cells (right) incubated with *o*-BMVC. The arrow showed the long decay time.

(a)


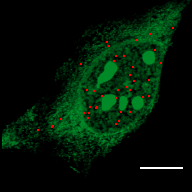

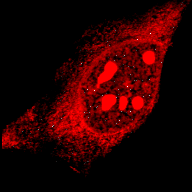


HeLa

(b)


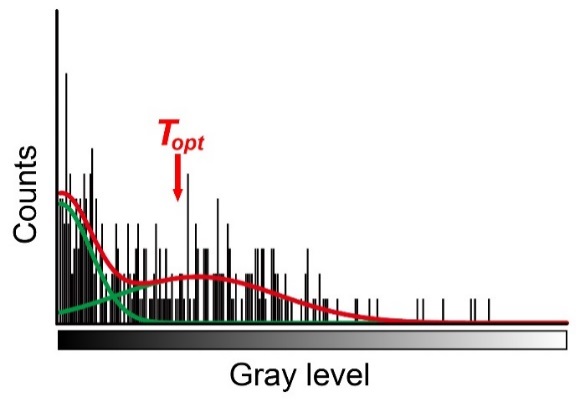


**Figure S3.** Time-gated FLIM imaging of *o*-BMVC foci in HeLa cancer cells after fixation with 70 % ethanol. The FLIM images of fixed cells (a, left) were presented in pseudocolor and were separated into two components with color in white (decay time ≥2.4 ns) and in red (decay time <2.4 ns). The Otsu threshold method is used to find an optimal threshold (*T_opt_*) to separate two clusters or the mixture of Gaussians in the longer lifetime channel. Typical gray-level histograms of fixed cells (b) of the longer lifetime (≥2.4 ns) channel can be fit as the mixture of Gaussians. The green lines are the Gaussian fitting curves and the red lines are the combination of fitting curves. Using the Otsu threshold method for data analysis, the weak signals can be eliminated, while the stronger signals can be preserved. The analyzed binary images of fixed cells (a, right) were presented in pseudocolor and were separated into two components with color in red (decay time ≥2.4 ns) and in green (decay time <2.4 ns). Scale bar: 10 μm.

(a)


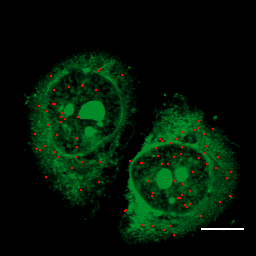

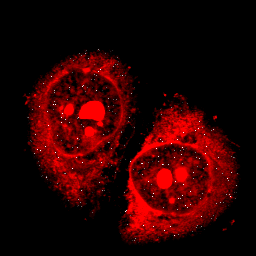


MCF-7/ADR

(b)


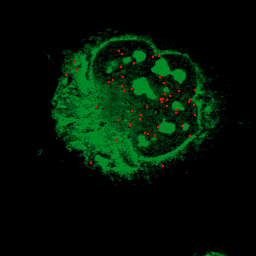

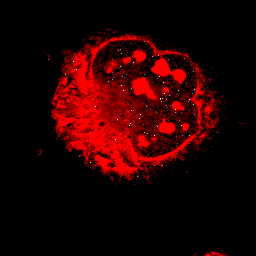


CL1-0


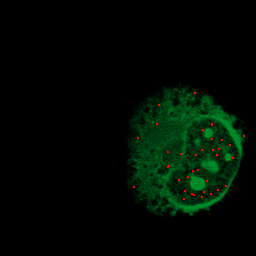

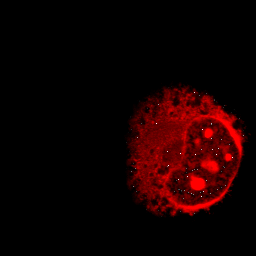
(c)

H1299


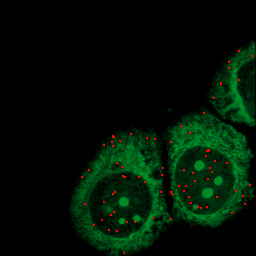

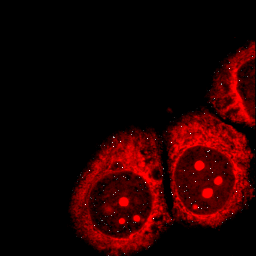
(d)

MCF-7


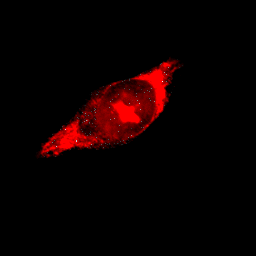

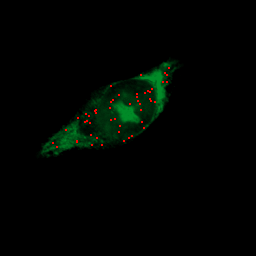
(e)

SAS

(f)


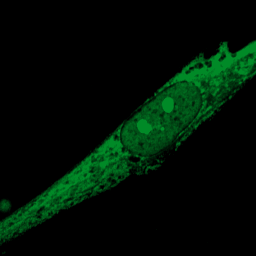

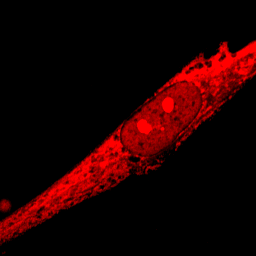


IMR-90


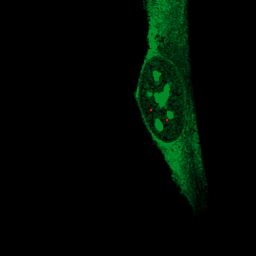

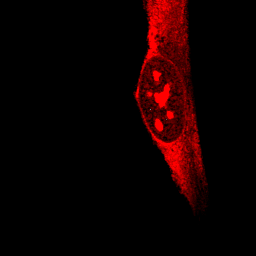
(g)

BJ

**Figure S4.** Time-gated FLIM imaging of original data (left) and analyzed data using the Otsu threshold method (right) for *o*-BMVC foci in MCF-7/ADR cancer cells (a), CL1-0 cancer cells (b), H1299 cancer cells (c), MCF-7 cancer cells (d), SAS cancer cells (e), IMR-90 normal cells (f) and BJ normal cells (g). Scale bar: 10 μm. The images were presented in pseudocolor and were separated into two components with color in white (decay time ≥2.4 ns) and in red (decay time <2.4 ns) for the original data, and in red (decay time ≥2.4 ns) and in green (decay time <2.4 ns) for the Otsu results.

(a) BF confocal


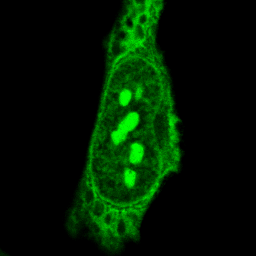

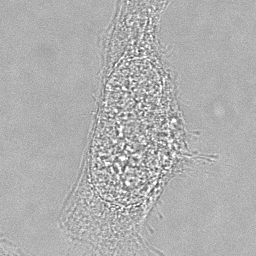


HeLa


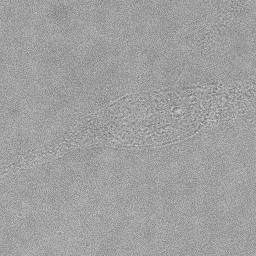

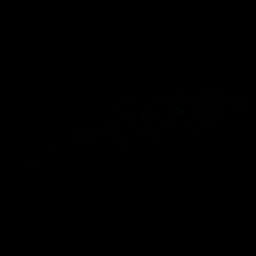


HeLa + DNase/RNase

(b) BF Otsu


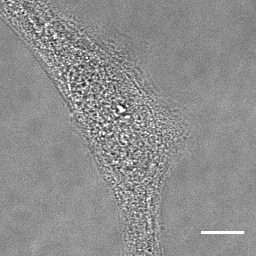

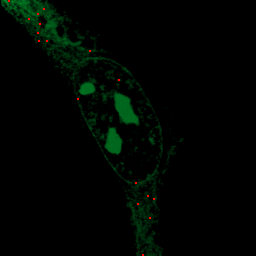


HeLa + DNase

**Figure S5.** The bright field (left) and confocal (right) images of HeLa cancer cells without (upper) and with (lower) DNase/RNase double pretreatment (a). Cells were fixed with 70 % ethanol for 10 min on coverslip and were treated with 20 μg/ml of DNase and RNase for 1 h at 37 °C followed by 5 µM *o*-BMVC staining for 10 min. Scale bar: 10 μm. The bright field (left) and Otsu (right) images of pre-treated HeLa cancer cells with DNase (b). Cells were fixed with 70 % ethanol for 10 min on coverslip and were treated with 20 μg/ml DNase for 1 h at 37 °C followed by 5 µM *o*-BMVC staining for 10 min.


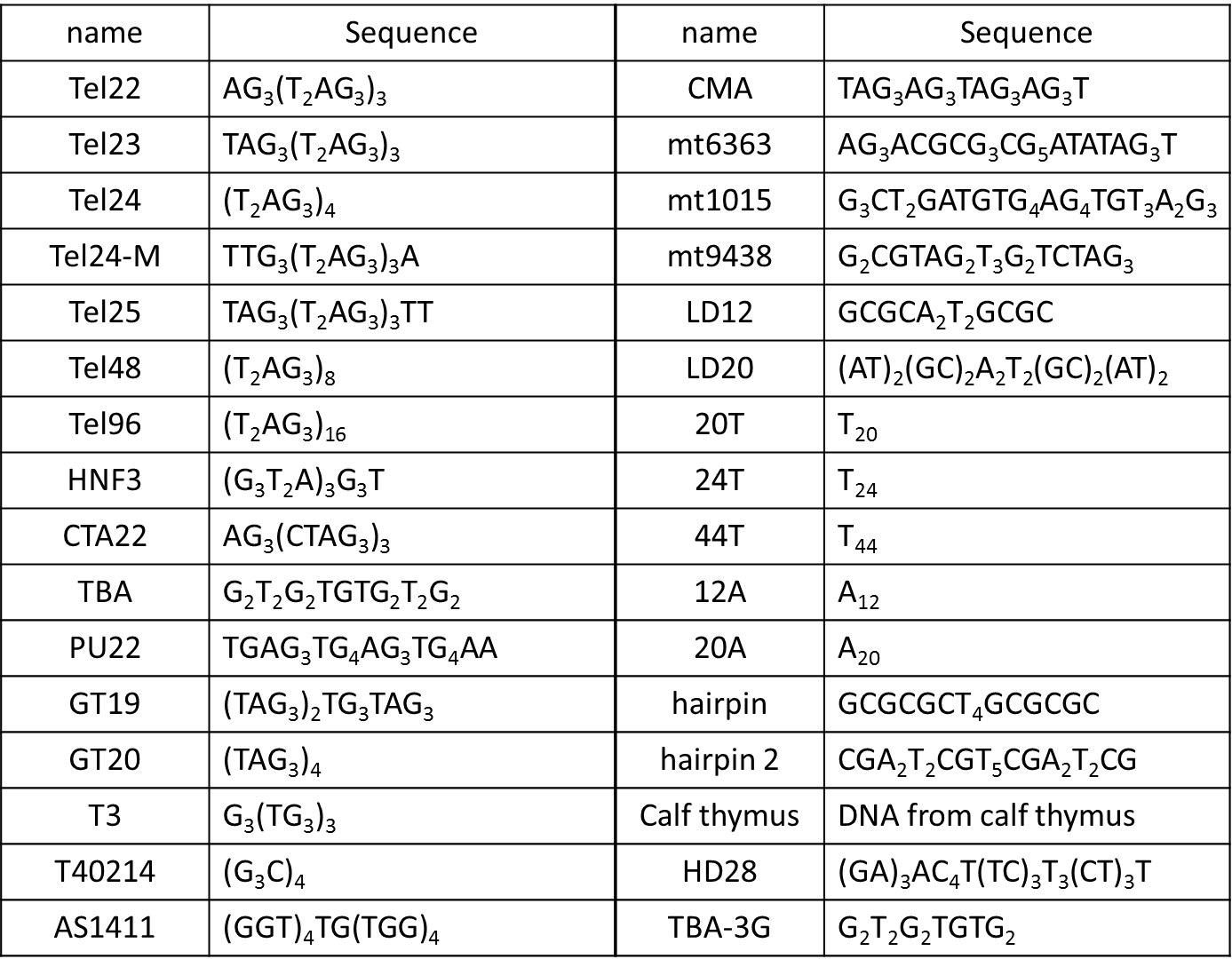


**Table S1.** Oligonucleotides studied in this work

| Patient number | Averaged number of Foci | Results of ο-BMVC test | Number of consistent result | Number of inconsistent result | Clinical diagnosis |
| --- | --- | --- | --- | --- | --- |
| 1 | 34.3 | + | 1 | 0 | Hypopharyngeal cancer |
| 2 | 17 | + | 1 | 0 | Gingival cancer |
| 3 | 21.3 | + | 1 | 0 | Tongue cancer |
| 4 | 13.9 | + | 1 | 0 | Buccal cancer |
| 5 | 53.5 | + | 1 | 0 | Buccal cancer |
| 6 | 16.5 | + | 1 | 0 | Tonsil cancer |
| 7 | 14.8 | + | 1 | 0 | Palate cancer |
| 8 | 34.6 | + | 1 | 0 | Buccal cancer |
| 9 | 27.2 | + | 1 | 0 | Buccal cancer |
| 10 | 9.8 | + | 1 | 0 | Tongue cancer |
| 11 | 20.5 | + | 1 | 0 | Gingival cancer |
| 12 | 18.2 | + | 1 | 0 | Tongue cancer |
| 13 | 11 | + | 1 | 0 | Tongue cancer |
| 14 | 20.4 | + | 1 | 0 | Buccal cancer |
| 15 | 16.1 | + | 1 | 0 | Tongue cancer |
| 16 | 13.7 | + | 1 | 0 | Tongue cancer |
| 17 | 17.4 | + | 1 | 0 | Buccal cancer |
| 18 | 23.6 | + | 1 | 0 | Tongue cancer |
| 19 | 19.1 | + | 1 | 0 | Mouth floor cancer |
| 20 | 17.8 | + | 1 | 0 | Buccal cancer |
| 21 | 18.3 | + | 1 | 0 | Buccal mucosa squamous cell carcinoma |
| 22 | 30.4 | + | 1 | 0 | Tongue cancer |
| 23 | 17.9 | + | 1 | 0 | Tongue cancer |
| 24 | 12 | + | 1 | 0 | Buccal cancer |
| 25 | 42.4 | + | 1 | 0 | Buccal cancer |
| 26 | 29 | + | 1 | 0 | Tongue cancer |
| 27 | 1.9 | - | 0 | 1 | Tongue cancer |
| 28 | 47.9 | + | 1 | 0 | Soft palate cancer |
| 29 | 25.7 | + | 1 | 0 | Gingival cancer |
| 30 | 70.7 | + | 1 | 0 | Palatal cancer |
| 31 | 20.4 | + | 1 | 0 | Gingival cancer |
| 32 | 77.9 | + | 1 | 0 | Tongue cancer |
| 33 | 54.2 | + | 1 | 0 | Buccal cancer |
| 34 | 52.3 | + | 1 | 0 | Gingival cancer |
| 35 | 20.4 | + | 1 | 0 | Laryngeal cancer |
| 36 | 20.5 | + | 1 | 0 | Tongue cancer |
| 37 | 17.8 | + | 1 | 0 | Tongue cancer |
| 38 | 20.9 | + | 1 | 0 | Mouth floor cancer |
| 39 | 42.5 | + | 1 | 0 | Gingival cancer |
| 40 | 22.4 | + | 1 | 0 | Hypopharyngeal cancer |
| 41 | 14.7 | + | 1 | 0 | Palatal cancer |
| 42 | 24.5 | + | 1 | 0 | Tonsillar tumor |
| 43 | 30.5 | + | 1 | 0 | Gum cancer |
| 44 | 72.5 | + | 1 | 0 | Tongue cancer |
| 45 | 28 | + | 1 | 0 | Tongue cancer |
| 46 | 17.7 | + | 1 | 0 | Tongue cancer |
| 47 | 35.4 | + | 1 | 0 | Buccal cancer |
| 48 | 71.5 | + | 1 | 0 | Tongue cancer |
| 49 | 16.8 | + | 1 | 0 | Tongue base cancer |
| 50 | 37 | + | 1 | 0 | Buccal cancer |
| 51 | 1.8 | - | 1 | 0 | - |
| 52 | 1.5 | - | 1 | 0 | - |
| 53 | 0.9 | - | 1 | 0 | - |
| 54 | 3.2 | - | 1 | 0 | - |
| 55 | 1.9 | - | 1 | 0 | - |
| 56 | 3.4 | - | 1 | 0 | - |
| 57 | 1.9 | - | 1 | 0 | - |
| 58 | 4.1 |  | 1 | 0 |  |
| 59 | 2.6 | - | 1 | 0 | - |
| 60 | 2.8 | - | 1 | 0 | - |
| 61 | 2.5 | - | 1 | 0 | - |
| 62 | 2.4 | - | 1 | 0 | - |
| 63 | 2.1 | - | 1 | 0 | - |
| 64 | 1.7 | - | 1 | 0 | - |
| 65 | 2.6 | - | 1 | 0 | - |
| 66 | 2.2 | - | 1 | 0 | - |
| 67 | 2.2 | - | 1 | 0 | - |
| 68 | 1.4 | - | 1 | 0 | - |
| 69 | 1.1 | - | 1 | 0 | - |
| 70 | 1.1 | - | 1 | 0 | - |

**Table S2.** All the results from *o*-BMVC test together with those from cytologic examination in this work.
